# Supplementary material for: Causal interplay between lactose intolerance and gut microbiota: a combined bidirectional Mendelian randomization and in vivo validation study
Source: Front Nutr. 2026 Jun 1;13:1803337. doi: 10.3389/fnut.2026.1803337 (PMC13265576; doi:10.3389/fnut.2026.1803337)
Supplement: Supplementary file 1 [file Data_Sheet_1.zip › supplementary materials/Forward/leave one out cross validation/ebi-a-GCST90027840.finngen_R12_E4_LACTONAS.pdf]

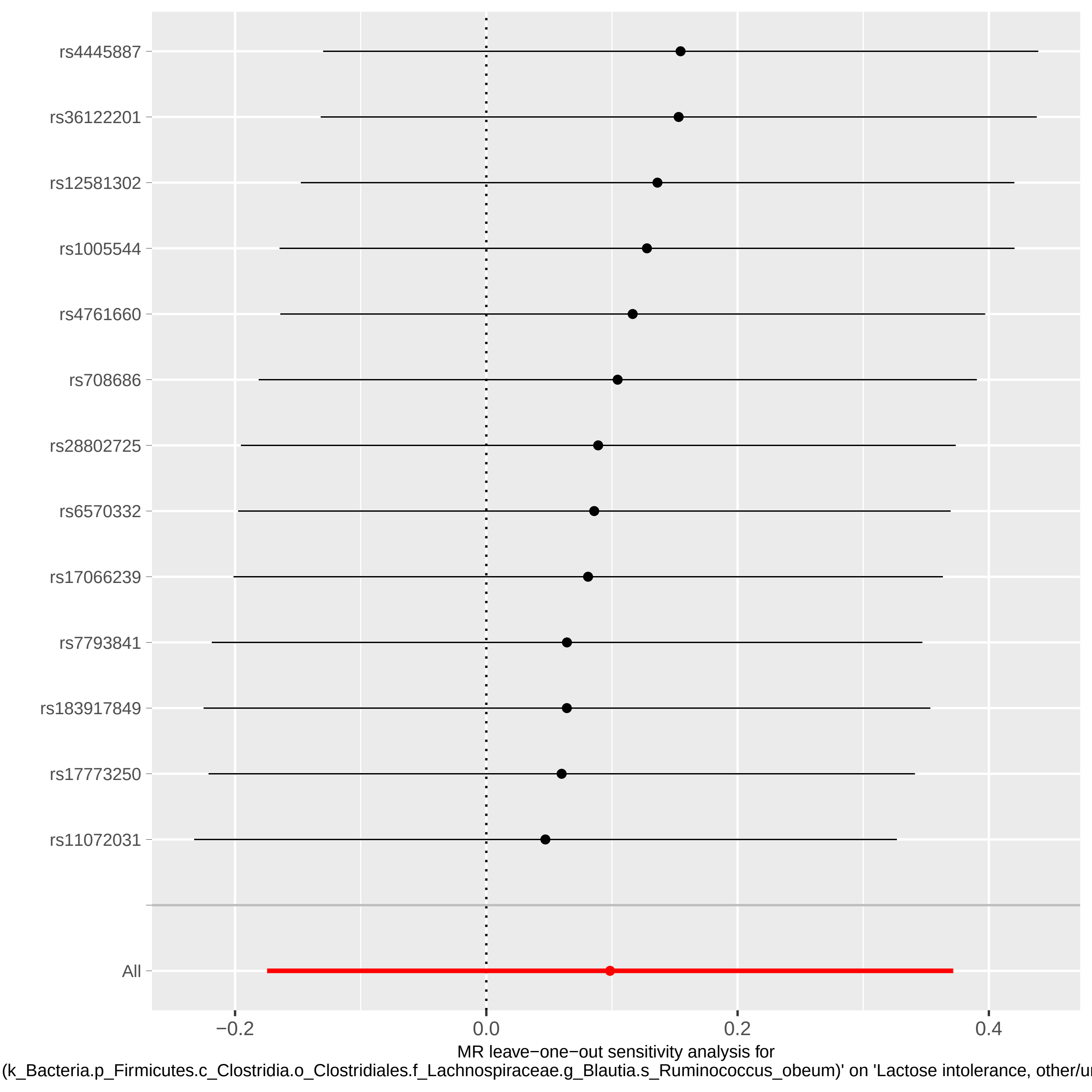
rs4445887
rs36122201
rs12581302
rs1005544
rs4761660
rs708686
rs28802725
rs6570332
rs17066239
rs7793841
rs183917849
rs17773250
rs11072031
All
−0.2
0.0
0.2
0.4
MR leave−one−out sensitivity analysis for
(k_Bacteria.p_Firmicutes.c_Clostridia.o_Clostridiales.f_Lachnospiraceae.g_Blautia.s_Ruminococcus_obeum)' on 'Lactose intolerance, other/u
